# Semaglutide in Patients with Obesity and Psoriasis: Insights from an 18-Month Real-World Experience

**DOI:** 10.3390/jcm15208015

**Published:** 2026-10-16

**Authors:** Ilaria Proietti, Concetta Potenza

**Affiliations:** UOC DERMATOLOGIA ‘Daniele Innocenzi’-ASL LATINA Sapienza Università di Roma-Polo Pontino, Corso della Repubblica, 79, 04100 Latina, Italy

**Keywords:** semaglutide, obesity, psoriasis, type 2 diabetes mellitus, real-world data

## Abstract

Psoriasis is often associated with obesity and type 2 diabetes mellitus (T2DM) due to a common low-grade chronic inflammatory mechanism amplified by metabolic dysfunction. Biologics have revolutionized psoriasis treatment, but patients with obesity show reduced responses and higher discontinuation rates. Glucagon-like peptide-1 receptor agonists—such as semaglutide—may enhance the efficacy of psoriasis care; however, data are limited in this context. We presented a retrospective analysis of our single-center real-world experience with 33 obese patients (24 men and 9 women) affected by mild (*n* = 19, 57.6%) or moderate (*n* = 14, 42.4%) psoriasis and treated with semaglutide over 18 months. Adherence to the semaglutide prescription was 94% (range 88–99%), with most patients (54.5%) having a last recorded dose of 1.0 mg. A significant mean reduction of 4.25 points in the PASI score (from 9.31 ± 1.54 to 5.05 ± 0.88; *p* < 0.001) was reported, followed by a mean decrease of 4.15 kg/m^2^ in BMI (from 32.53 ± 1.92 to 28.38 ± 1.59 kg/m^2^; *p* < 0.001) and significant improvements in all metabolic parameters. In addition, there was a significant decrease in inflammatory markers (CRP decreased from 5.47 ± 1.69 to 2.69 ± 0.94 mg/L, and ESR from 17.76 ± 4.22 to 11.64 ± 2.83 mm/h). More than fifty-seven percent (57.6%) of patients experienced mild and transitory gastrointestinal reactions, but no cases of discontinuation or cutaneous reaction occurred. Data provide additional insights into the role of semaglutide in patients with mild-to-moderate psoriasis. Large-scale, multicenter studies are needed to validate long-term results.

## 1. Introduction

Psoriasis is a chronic inflammatory condition of the skin and an immune-mediated disease characterized by different phenotypic variants—with plaque psoriasis being the most frequent variant. The pro-inflammatory nature of the disease may be further amplified by metabolic factors, contributing to keratinocyte hyperproliferation [1,2,3,4]. Frequently associating with psoriasis, obesity and type 2 diabetes mellitus (T2DM) should not be regarded merely as comorbidities, as they share common inflammatory mechanisms involving metabolic dysfunction, adipokine imbalance, and chronic low-grade inflammation [1,2,3,4]. Recent evidence highlights that the adipose tissue sustains the immunologic activity by triggering pro-inflammatory cytokine and adipokines production, which promotes chronic systemic inflammation. This results in insulin resistance and ectopic fat deposition, increasing cardiovascular (CV) risk and worsening metabolic conditions [2,3].

Biologic therapies have revolutionized the treatment of psoriasis by enabling high rates of skin clearance. However, patients with psoriasis and concomitant obesity have been shown to exhibit a reduced response to biologic therapies and, consequently, higher treatment discontinuation rates [4]. In addition, cardiovascular risk increases in these subjects, in parallel with psoriasis severity and metabolic burden, exposing them to substantial long-term health risks [2,3].

Weight management has therefore become an important component of psoriasis care, as both treatments for obesity and antidiabetic drugs have been associated with improved efficacy of psoriasis therapies [2,4]. In particular, increasing attention has been directed toward metabolic-targeted molecules modulating both metabolic dysfunction and inflammatory pathways involved in psoriatic disease [2,3,5,6,7]. Among them, the weight-loss properties of Glucagon-like peptide-1 receptor agonists (GLP-1RAs), particularly semaglutide, have driven significant clinical interest in recent years in dermatological practice, and particularly in the treatment of psoriasis. Despite some adverse skin reactions, several reviews have confirmed the potential dermatological effects of GLP-1RAs as mediated by immunomodulatory benefits [5,6,7,8,9,10]. From a metabolic perspective, these medications activate GLP-1 receptors to enhance insulin secretion while reducing glucagon release and slowing down gastric emptying, resulting in better glycemic control, reduced appetite, and substantial weight loss. Moreover, treatment with GLP-1RAs has been shown to decrease CV risk [6,7]. A reduction in body weight, along with the potential interaction of GLP-1RAs with signaling pathways involved in inflammation and immune response, may contribute to a reduction in pro-inflammatory adipokines involved in systemic and cutaneous inflammation. These mechanisms could potentially favor improvements in cutaneous manifestations of psoriasis [7,9,11].

Given the promising impact of GLP-1RAs on the treatment of inflammatory skin conditions, this approach is receiving progressively greater attention. However, only a limited number of case reports and a few preliminary prospective studies have been published on this topic to date [5,10,12], leading to a consistent call for further evidence. With the aim of expanding the body of clinical evidence, we present a retrospective analysis of our single-center real-world experience with 33 obese patients with psoriasis treated with semaglutide over an 18-month period.

## 2. Materials and Methods

Data from patients with mild or moderate psoriasis and treated with the two injectable, commercially available semaglutides (for obesity and T2DM) between July 2022 and February 2026 were collected and analyzed. Psoriasis was defined as mild if Psoriasis Area and Severity Index (PASI) ≤ 10, Body Surface Area (BSA) ≤ 10%, and Dermatology Life Quality Index (DLQI) ≤ 10, and moderate if PASI > 10 e/o BSA > 10% e/o DLQI > 10.

Patients were eligible for the analysis if they had obesity (body mass index, BMI ≥ 30 kg/m^2^) with at least one weight-related comorbidity, were naïve to semaglutide at baseline, and had at least 18 months of follow-up. According to our center’s prescribing criteria, patients receiving semaglutide were also required to have initiated a lifestyle modification program, including diet and exercise, and to have no contraindications to semaglutide treatment. All patients started semaglutide therapy with a dose of 0.25 mg; dose escalation was considered every four weeks according to glycemic and weight-control targets and individual treatment tolerability.

Some patients had previously received systemic or biologic therapies, as reported in Table 1; however, based on clinical judgment, these treatments had been discontinued before baseline, mainly because of inadequate clinical response, inability to maintain conventional systemic treatments (e.g., cyclosporine) due to their metabolic profile or relative contraindications, or limited access to biologic therapies. No predefined wash-out period was applied, given the retrospective, real-world nature of the study. However, none of the included patients received concomitant systemic or biologic therapies for psoriasis during the 18-month semaglutide observation period.

Information retrieved included psoriasis duration, clinical phenotype, anatomical sites involved, presence of psoriatic arthritis, and presence of comorbidities such as hypertension, T2DM and Non-Alcoholic Fatty Liver Disease (NAFLD). Any previous dermatological treatment was recorded. Changes in the frequency and potency of topical therapies during follow-up could not be systematically assessed from the available retrospective clinical records.

To evaluate any improvement in the skin condition and metabolic health, the following values were measured at the beginning of the study and after 18 months: BMI, psoriasis severity assessed by the Psoriasis Area and Severity Index, metabolic parameters (fasting blood glucose, insulin, Homeostatic Model Assessment of Insulin Resistance [HOMA-IR], triglycerides and total cholesterol), inflammatory markers (C-reactive protein [CRP] and erythrocyte sedimentation rate [ESR]) and additional biochemical parameters (urea and serum uric acid). These latter parameters were included as part of the overall metabolic and biochemical assessment of this population with obesity, rather than as specific biomarkers of psoriasis severity.

Adherence to semaglutide treatment was calculated for each patient by considering the number of weekly semaglutide injections that were administered divided by the scheduled number of injections for the individual period of observation, multiplied by 100. The resultant data were then subjected to analysis as a continuous percentage.

### 2.1. Statistical Analysis

Data extracted from the institutional electronic medical records were deidentified and exported to Microsoft Excel™ (version 2609 Build 16.0.20430.20032) for descriptive statistical analysis. Normality was assessed using a combination of skewness and kurtosis measures, the Shapiro–Wilk test, and visual inspection of Q–Q plots and box plots. Categorical values are reported as absolute and relative frequencies (*n*, %), whereas quantitative variables are expressed as mean ± standard deviation (SD). The paired *t*-test was used for comparison of mean values between baseline and 18-month follow-up matching samples. Moreover, explorative analyses were conducted: longitudinal changes in quantitative variables between baseline and the 18-month follow-up were investigated through linear mixed models (LMMs) with subjects as random effects. The multivariate models incorporated time and selected clinical and demographic covariates (sex, age, adherence percentage, weekly dose, years of psoriasis duration, occurrence of T2D or NAFLD) to derive adjusted estimates. Weekly dose as well as occurrence of T2D or NAFLD were entered as categorical variables. Given the limited sample size relative to the number of model parameters, these adjusted models were considered as purely exploratory and hypothesis-generating rather than confirmatory. Accordingly, estimates for individual covariates were interpreted cautiously because of the potential for imprecision and model overfitting. The results were expressed as regression coefficients with 95% confidence intervals (95% CI) and *p*-values. The goodness of fit of the models was described using marginal R^2^ and conditional R^2^; *p* < 0.05 was considered to be statistically significant. LMM analysis was conducted in R (version 4.4.1).

### 2.2. Ethical Considerations

This retrospective analysis was conducted in accordance with the principles of the Declaration of Helsinki and applicable institutional and regulatory requirements. Only de-identified data collected as part of routine clinical care were analyzed, and no additional procedures, interventions, or direct patient contact were undertaken for research purposes.

## 3. Results

In this retrospective case series, we identified a total of 33 patients (24 men and 9 women) with mild (*n* = 19, 57.6%) or moderate (*n* = 14, 42.4%) psoriasis. The median age at first visit was 47 years (range 33–62 years), and the median time of disease was 14 years (range 2–27 years). The most common phenotype was plaque psoriasis, present in 81.8% cases; a minority of cases presented with guttate, palmoplantar, or inverse psoriasis (*n* = 2 for each condition, 6.1%). Mean baseline PASI score was 9.3 ± 1.5. All patients had obesity (BMI ≥ 30, mean 32.53 ± 1.92 kg/m^2^, range 30.00–36.20) and about half of them had hypertension (*n* = 14, 42.4%), T2DM (*n* = 15, 45.5%) and NAFLD (*n* = 19, 57.6%), but no cases reported CV events, inflammatory or acute infective disease, or active neoplasms. All baseline demographic and clinical characteristics are summarized in Table 1.

During the 18-month treatment period, the median adherence to the semaglutide prescription was 94% (range 88–99%). The last recorded dose was 0.5 mg in 9 subjects (27.3%), 1.0 mg in 18 patients (54.5%), and 2.4 mg in 6 patients (18.2%). Overall, after 18 months, a significant mean reduction of 4.25 points in the PASI score (from 9.31 ± 1.54 to 5.05 ± 0.88; *p* < 0.001) was observed, and all patients with baseline moderate psoriasis passed to a mild condition. Likewise, a statistically significant improvement of all anthropometric and metabolic parameters was observed (each *p* < 0.001, Table 2).

In particular, BMI showed a mean decrease of 4.15 kg/m^2^, dropping to a mean value of 28.38 kg/m^2^; blood glucose decreased from 111.88 ± 8.55 to 99.06 ± 8.93 mg/dL, and insulin from 20.55 ± 3.75 to 13.84 ± 2.63 µIU/mL; HOMA-IR decreased of 2.29 points, triglyceride levels fell to normal mean values of 140.45 ± 20.15 mg/dL, and total cholesterol decreased about 12%, falling within the normal range (mean value 192.12 ± 16.74 mg/dL). Urea showed a modest decrease from 17.39 ± 2.63 to 16.33 ± 2.69 mg/dL (−6.3%), whereas uric acid levels exhibited a more pronounced reduction (−16.2%), decreasing from 6.84 ± 0.72 to 5.73 ± 0.76 mg/dL. In addition, there was a significant decrease in inflammatory markers, with CRP decreasing from 5.47 ± 1.69 to 2.69 ± 0.94 mg/L and ESR decreasing from 17.76 ± 4.22 to 11.64 ± 2.83 mm/h.

In the linear mixed multivariate models, time was the only predictor significantly associated with BMI, triglycerides, total cholesterol, CRP, ESR and urea (Table S1). These outcomes were found to be significantly reduced after 18 months compared with baseline, regardless of the other covariates. Time was significantly associated also with PASI score (Table S1); moreover, the model showed a positive association between PASI and weekly semaglutide dose: compared with patients receiving 0.5 mg, those receiving 1 mg had a 1.00-point higher mean PASI score (95% CI: 0.01–2.00; *p* = 0.049), whereas those receiving the highest dose had a 2.01-point higher mean PASI score (95% CI: 0.49–3.53; *p* = 0.011). This finding should be interpreted cautiously, as it may reflect a confounding by indication effect: rather than a dose-dependent worsening of PASI, this result could indicate that patients with greater disease severity at baseline could have more likely received higher semaglutide doses.

The presence of NAFLD was significantly associated with increased levels of blood glucose (+7.85 mg/dL; 95% CI: 0.38–15.33; *p* = 0.040), higher insulin levels (+2.78 µIU/mL, 95% CI: 0.00–5.57; *p* = 0.050, borderline) and higher HOMA-IR values (+1.04, 95% CI: 0.30–1.79; *p* = 0.007) (Table S1). Conversely, treatment adherence was found to be associated with decreased levels of blood glucose (−1.39 mg/dL, 95% CI: −2.71 to −0.08; *p* = 0.038) and HOMA-IR (−0.14, 95% CI: −0.27 to −0.01; *p* = 0.035). Finally, males had significantly higher mean serum uric acid levels than females, with a mean difference of +0.62 mg/dL (95% CI: 0.04–1.21; *p* = 0.038).

As shown in Table 3, 57.6% of patients experienced mild and transitory gastrointestinal reactions to semaglutide treatment. In six cases (18.2%), the symptoms persisted for up to 18 months. In such cases, the administration of medication for constipation or reflux was provided, and no instances of discontinuation were documented. No cases of cutaneous reactions occurred.

## 4. Discussion

This retrospective analysis on a series of 33 cases further provides clinical evidence of the therapeutic effect of semaglutide in patients with obesity and mild to moderate psoriasis. After 18 months, subjects receiving semaglutide showed improvements in anthropometric and metabolic parameters, together with a 4.25-point reduction in PASI score.

Semaglutide belongs to the class of GLP-1RAs, which were initially indicated for patients with T2DM and obesity due to their ability to maintain glycemic control without increasing the risk for hypoglycemia [13]. More recently, GLP-1RAs have gained interest due to their potential role in the management of psoriasis disease. Indeed, these molecules have been shown to reduce local or systemic inflammation [6,9,11,14]. Considering that chronic low-grade inflammation is a prevalent pathological feature of obesity, psoriasis and several other chronic conditions (e.g., non-alcoholic steatohepatitis, atherosclerosis, neurodegenerative disorders, diabetic nephropathy and asthma) [15,16], research on GLP-1RAs is still ongoing in an attempt to establish the efficacy and safety of these molecules in the treatment of inflammatory diseases [11,17,18]. However, clinical evidence on psoriasis is fragmented, and this is particularly true for semaglutide, making it unclear what phenotypic criteria should be used for prescription [19]. Moreover, it is important to note that the dermatological effects may vary among different GLP-1RA agents. Therefore, a more detailed analysis of these molecules is recommended in order to explore different profiles in terms of cutaneous improvement, systemic benefit, tolerability, or paradoxical cutaneous reactions [5].

While current psoriasis management guidelines, such as those from the American Academy of Dermatology (AAD) and the National Psoriasis Foundation (NPF), do not yet formally include GLP-1RAs as a direct treatment option, they strongly advocate for body weight control in patients with overweight or obesity due to the established link with psoriasis severity [4,8]. Indeed, weight reduction has been demonstrated to have a positive impact on the severity of this skin disease [20], and this may be the result of an improvement of systemic inflammatory conditions [1,7,21,22].

In our cohort, injectable semaglutide treatment resulted in a statistically significant mean BMI reduction of 4.15 kg/m^2^, with a mean percentage reduction ranging from 10% to 18%. This reduction was accompanied by favorable changes in anthropometric, metabolic and inflammatory parameters. Most patients received a dosage of 1 mg, while approximately 18% reached a 2.4 mg dose. Notably, the parallel reduction in biochemical markers (CRP and ESR), together with improvements in BMI, metabolic parameters, and PASI, may reflect an overall improvement in obesity-related low-grade systemic inflammation [23]. However, our study cannot distinguish a potential direct anti-inflammatory effect of semaglutide from the effects associated with weight loss and metabolic improvement. In line with our findings, few case reports describing the use of semaglutide in patients with psoriasis and T2DM or obesity have reported improvements in metabolic parameters and psoriasis severity, although these cases generally involved patients with more severe psoriasis at baseline than those included in our cohort. In particular, Lin et al. described a pronounced reduction in CRP (from 17.3 to 2.7 mg/L), improved insulin resistance (HOMA-IR: from 5.74 to 4.08) and enhanced psoriasis condition (PASI: from 22 to 6.2) in a 49-year-old subject with psoriasis and concurrent T2DM and MASLD treated with semaglutide (maintenance dose of 1 mg) for 12 months [24]. Using the same doses of the medication, Malavazos et al. recorded a 98% reduction in PASI score in a 50-year-old obese woman with T2DM, alongside a 25% decrease in BMI, 24% in fasting glucose, 11% in total cholesterol and 74% in HOMA-IR. The inflammatory parameters of CRP and ESR decreased by 88% and 43%, respectively [16]. Consistently, a recent real-world study by Conforti et al. in patients with knee osteoarthritis and overweight/obesity reported concurrent reductions in body weight and BMI (about 11% each), as well as CRP (approximately 43.9%) and ESR (approximately 30.0%), after six months of semaglutide treatment, suggesting a possible improvement in obesity-associated systemic inflammation, although the different clinical setting and observational design preclude direct comparison and causal interpretation [25]. Costanzo et al. reported the case of a 73-year-old male patient with class III obesity (BMI: 42.7 kg/m^2^), T2DM and severe psoriasis (PASI: 33.2) who improved his glycemic and obesity parameters after 10 months of semaglutide therapy at the maintenance dose of 1 mg in addition to metformin, reducing BMI by 10% and PASI by 92% [15]. However, the magnitude of PASI reduction reported in these case reports should be interpreted in light of their substantially higher baseline disease severity (PASI 22–33.2).

In addition to these case reports, results from two prospective studies align with our findings. Although the authors acknowledge that both were limited by sample sizes and relatively short follow-up, these studies could further indicate a positive effect of injected semaglutide in patients with T2DM and psoriasis. The first refers to a randomized clinical trial involving 16 patients with psoriasis and T2DM treated with semaglutide 1.0 mg/patient/week over a period of 12 months; the authors reported a significant reduction in BMI (from 33.0 to 30.7 kg/m^2^), PASI (from 21 to 10) and CRP (from 3.8 to 1.9) [23]. Likewise, a short-term (6 months) prospective cohort study on 43 patients with obesity and psoriasis, receiving weekly semaglutide along with lifestyle counselling, confirmed significant reductions in PASI (−48%), weight (−11.8%) and BMI (−11.4%), with improved metabolic markers [26]. Of note, the latter is the sole publication to report a substantial number of patients and to have undertaken a correlation analysis between PASI improvement and baseline variables. Baseline disease severity, depressive symptoms, insulin resistance and preperitoneal fat were negatively associated with PASI improvement, leading to poorer clinical response, while BMI and glycemic markers were not significantly associated after adjustment [26]. In our retrospective series, an association was also observed between PASI score and the weekly dose of semaglutide, with higher PASI scores observed among patients receiving higher final doses. However, this finding should not be interpreted as evidence of a dose-dependent effect of semaglutide on psoriasis severity. Rather, it most likely reflects confounding by indication, as patients with greater clinical severity received higher semaglutide doses (Figure S1). Therefore, no direct conclusions regarding the relationship between semaglutide dose and psoriasis efficacy can be drawn from this analysis. What is more, high adherence to the treatment resulted in an associated improvement of blood glucose and HOMA-IR score. Instead, the baseline NAFLD condition was observed to exert a detrimental influence on blood glucose, insulin, and HOMA-IR levels in comparison with patients without this condition.

A salient point of discussion is the prevalence of comorbidities in cases of psoriasis. The therapeutic protocols currently involve polypharmacy, which carries an elevated risk of adverse events. In cases involving the presence of multiple comorbidities, the pleiotropic therapeutic effects of semaglutide have been suggested to potentially contribute to simplifying treatment regimens [24]. This potential benefit was not specifically assessed in our study and requires further investigation. However, incorporating these interventions into a multidisciplinary management strategy encompassing conventional dermatological therapies, dietary interventions, and lifestyle modifications would align with the prevailing recommendations that underscore the value of comprehensive comorbidity assessment and holistic patient care in psoriasis management [8].

Overall, the present case series provide additional insights into the role of semaglutide in patients with mild to moderate psoriasis. However, the analysis presents several limitations. First, results should be interpreted only as preliminary evidence for the use of semaglutide as an adjunctive agent to first-line topical therapies. In this context, results from LMMs should be considered purely exploratory and hypothesis-generating. The single-center retrospective design and the absence of a comparator group represent major limitations; therefore, the authors suggest caution in directly attributing to semaglutide the observed improvement in the PASI score. Moreover, the uncontrolled and unblinded design does not allow potential placebo or Hawthorne effects to be excluded. Given the retrospective real-world design, no predefined wash-out period from previous systemic or biologic psoriasis treatments was applied, and changes in the frequency or potency of topical therapies could not be systematically assessed. Other limitations include the low sample size, potential confounding by indication in the output of LMM analyses, possible overparameterization of the LMMs, lack of systematic assessment of adherence to dietary and physical activity interventions, and the lack of follow-up beyond 18 months.

Future large-scale, multicenter, randomized controlled studies are needed to validate the long-term efficacy and safety of semaglutide in patients with mild to moderate psoriasis.

## Figures and Tables

**Table 1:** Baseline demographic and clinical characteristics of the study population (*n* = 33). Data are presented as mean ± standard deviation (SD), median (range), or number (%), as appropriate. Percentages were calculated using the total study population (*n* = 33). Participants could present with more than one anatomical site of involvement; therefore, percentages for anatomical sites do not sum to 100%.

Characteristic	Value
**Participants, *n***	33
**Sex, *n* (%)**	
Female	9 (27.3)
Male	24 (72.7)
**Age, years**	48.1 ± 7.7
Median (range)	47 (33–62)
**Hypertension, *n* (%)**	14 (42.4)
**T2DM, *n* (%)**	15 (45.5)
**NAFLD, *n* (%)**	19 (57.6)
**Psoriasis duration, years**	13.7 ± 7.2
Median (range)	14 (2–27)
**Psoriatic arthritis, *n* (%)**	10 (30.3)
**Psoriasis phenotype, *n* (%)**	
Plaque psoriasis	27 (81.8)
Guttate psoriasis	2 (6.1)
Inverse psoriasis	2 (6.1)
Palmoplantar psoriasis	2 (6.1)
**Anatomical sites involved, *n* (%)**	
Trunk	17 (51.5)
Scalp	10 (30.3)
Limbs	8 (24.2)
Elbows	8 (24.2)
Knees	8 (24.2)
Palms	3 (9.1)
Soles	3 (9.1)
Flexural areas	2 (6.1)
Axillae	2 (6.1)
Groin	2 (6.1)
**Previous psoriasis treatment, *n* (%)**	
Topical therapy + NB-UVB	9 (27.3)
Topical therapy	8 (24.2)
Topical corticosteroids + calcipotriol	5 (15.2)
Apremilast	3 (9.1)
Acitretin	2 (6.1)
Anti-IL-17 biologic	2 (6.1)
Anti-TNF biologic	2 (6.1)
Anti-IL-23 biologic	1 (3.0)
Cyclosporine	1 (3.0)
Phototherapy	0 (0.0)

Abbreviations: T2DM, Type 2 Diabetes Mellitus; NAFLD, Non-Alcoholic Fatty Liver Disease; NB-UVB, narrowband ultraviolet B phototherapy; IL, interleukin; TNF, tumor necrosis factor.

**Table 2:** Changes in clinical, anthropometric, metabolic, and inflammatory parameters from baseline to 18 months. Data are presented as mean ± standard deviation (SD). Mean differences were calculated as the 18-month value minus the baseline value and are reported with 95% confidence intervals (95% CI). Comparisons were performed using paired-samples Student’s *t*-tests. Statistical significance was defined as *p* < 0.05.

Outcome	Baseline, Mean ± DS	18 Months, Mean ± DS	Mean Difference (CI 95%)	*p* Value
**BMI (kg/m^2^)**	32.53 ± 1.92	28.38 ± 1.59	−4.15 (−4.41; −3.88)	<0.001
**PASI**	9.31 ± 1.54	5.05 ± 0.88	−4.25 (−4.71; −3.80)	<0.001
**Fasting glucose (mg/dL)**	111.88 ± 8.55	99.06 ± 8.93	−12.82 (−13.95; −11.68)	<0.001
**Insulin (µIU/mL)**	20.55 ± 3.75	13.84 ± 2.63	−6.72 (−7.61; −5.82)	<0.001
**HOMA-IR**	5.68 ± 1.10	3.39 ± 0.73	−2.29 (−2.55; −2.03)	<0.001
**Triglycerides (mg/dL)**	194.97 ± 25.42	140.45 ± 20.15	−54.52 (−61.08; −47.95)	<0.001
**Total cholesterol (mg/dL)**	217.91 ± 19.97	192.15 ± 16.74	−25.76 (−28.59; −22.93)	<0.001
**Blood urea (mg/dL)**	17.39 ± 2.63	16.33 ± 2.69	−1.06 (−1.38; −0.74)	<0.001
**Uric acid (mg/dL)**	6.84 ± 0.72	5.73 ± 0.76	−1.11 (−1.27; −0.95)	<0.001
**CRP (mg/L)**	5.47 ± 1.69	2.69 ± 0.94	−2.78 (−3.19; −2.36)	<0.001
**ESR (mm/h)**	17.76 ± 4.22	11.64 ± 2.83	−6.12 (−7.07; −5.18)	<0.001

Abbreviations: BMI, body mass index; PASI, Psoriasis Area and Severity Index; HOMA-IR, Homeostasis Model Assessment of Insulin Resistance; CRP, C-reactive protein; ESR, erythrocyte sedimentation rate.

**Table 3:** Semaglutide exposure, treatment adherence, and gastrointestinal safety outcomes. Data are presented as mean ± standard deviation (SD), median (range), or number (%), as appropriate. Percentages were calculated using the total study population (*n* = 33). Semaglutide dose refers to the maintenance dose achieved at the 18-month follow-up.

Characteristic	Value
**Semaglutide dose at 18 months, *n* (%)**	
0.5 mg	9 (27.3)
1.0 mg	18 (54.5)
2.4 mg	6 (18.2)
**Treatment adherence (%)**	94.1 ± 2.7%
Median (range)	94 (88–99)
**Gastrointestinal adverse events, *n* (%)**	
None	14 (42.4)
Mild nausea	7 (21.2)
Constipation	5 (15.2)
Moderate nausea	4 (12.1)
Dyspepsia	1 (3.0)
Occasional vomiting	1 (3.0)
Mild diarrhea	1 (3.0)

## Data Availability

Data are available upon reasonable request.

## Supplementary Materials

The following supporting information can be downloaded at https://www.mdpi.com/article/10.3390/jcm15208015/s1, Figure S1: Predicted PASI scores at baseline and after 18 months according to weekly dose. Points represent model-estimated marginal means, and error bars indicate 95% confidence intervals. Estimates were obtained from a linear mixed-effects model with a random intercept for patient; Table S1 Changes in quantitative measures from baseline to 18 months evaluated through the mixed-effects regression models. Values are reported as regression coefficients (95% CI); *p*-value. Marginal R^2^ represents the variance explained by fixed effects, whereas conditional R^2^ represents the variance explained by both fixed and random effects (i.e., the subjects in the model). Bold values indicate statistical significance (*p* ≤ 0.05).

## Abbreviations

The following abbreviations are used in this manuscript:

T2DM	Type 2 Diabetes Mellitus
CV	Cardiovascular
GLP-1RAs	Glucagon-Like Peptide-1 Receptor Agonists
NAFLD	Non-Alcoholic Fatty Liver Disease
BMI	Body Mass Index
CRP	C-Reactive Protein
ESR	Erythrocyte Sedimentation Rate
SD	Standard Deviation
LMM	Linear Mixed Models
CI	Confidence Intervals
AAD	Academy Of Dermatology
NPF	National Psoriasis Foundation
MEN2 syndrome	Multiple Endocrine Neoplasia Type 2
