# Supplementary material for: Semaglutide in Patients with Obesity and Psoriasis: Insights from an 18-Month Real-World Experience
Source: J Clin Med. 2026 Oct 16;15(20):8015. doi: 10.3390/jcm15208015 (PMC13705784; doi:10.3390/jcm15208015)

Supplementary Material

Table S1. Changes in quantitative measures from baseline to 18 months evaluated through the mixed-effects regression models. Values are reported as regression coefficients (95% CI); p-value. Marginal R^2 represents the variance explained by fixed effects, whereas conditional R^2 represents the variance explained by both fixed and random effects (i.e., the subjects in the model). Bold values indicate statistical significance (p ≤ 0.05).

PREDICTOR	BMI	PASI	GLUCOSE	INSULIN	HOMA-IR	TG	TC	CRP	ESR	UREA	SUA
Time (18 months vs baseline)	**−4.15 (−4.41, −3.88); <0.001**	**−4.25 (−4.70, −3.81); <0.001**	**−12.82 (−13.93, −11.70); <0.001**	**−6.72 (−7.60, −5.83); <0.001**	**−2.29 (−2.54, −2.03); <0.001**	**−54.52 (−60.97, −48.06); <0.001**	**−25.76 (−28.55, −22.97); <0.001**	**−2.78 (−3.19, −2.36); <0.001**	**−6.12 (−7.05, −5.19); <0.001**	**−1.06 (−1.37, −0.75); <0.001**	**−1.11 (−1.27, −0.95); <0.001**
Weekly dose: 1 mg	−0.71 (−2.47, 1.05); 0.421	**1.00 (0.01, 2.00); 0.049**	−0.89 (−8.77, 6.98); 0.821	1.90 (−1.03, 4.83); 0.199	0.43 (−0.35, 1.22); 0.274	0.64 (−20.74, 22.03); 0.952	13.85 (−1.37, 29.07); 0.074	0.00 (−1.38, 1.39); 0.997	0.13 (−3.42, 3.67); 0.943	−1.68 (−4.23, 0.88); 0.194	0.25 (−0.45, 0.94); 0.485
Weekly dose: 2 mg	−0.01 (−2.70, 2.67); 0.991	**2.01 (0.49, 3.53); 0.011**	1.80 (−10.20, 13.79); 0.765	1.25 (−3.21, 5.72); 0.576	0.39 (−0.81, 1.59); 0.519	3.06 (−29.52, 35.65); 0.851	−0.14 (−23.33, 23.05); 0.991	−0.15 (−2.26, 1.97); 0.891	−1.58 (−6.98, 3.83); 0.561	−0.36 (−4.25, 3.53); 0.854	−0.36 (−1.42, 0.71); 0.506
Psoriasis duration, years	0.03 (−0.06, 0.12); 0.512	−0.01 (−0.06, 0.04); 0.714	−0.08 (−0.50, 0.33); 0.688	−0.01 (−0.16, 0.15); 0.923	−0.01 (−0.05, 0.03); 0.750	−1.04 (−2.17, 0.09); 0.069	−0.73 (−1.53, 0.08); 0.075	0.01 (−0.06, 0.08); 0.757	−0.00 (−0.19, 0.18); 0.964	−0.03 (−0.16, 0.11); 0.696	−0.02 (−0.05, 0.02); 0.352
T2DM	−1.34 (−3.21, 0.53); 0.157	−0.27 (−1.32, 0.79); 0.615	5.66 (−2.68, 14.00); 0.180	−2.03 (−5.14, 1.08); 0.196	−0.29 (−1.12, 0.54); 0.487	5.90 (−16.76, 28.57); 0.604	12.85 (−3.28, 28.98); 0.116	0.28 (−1.19, 1.75); 0.708	−0.95 (−4.71, 2.81); 0.614	−0.78 (−3.49, 1.92); 0.564	−0.10 (−0.84, 0.64); 0.795
NAFLD	0.80 (−0.88, 2.47); 0.345	0.23 (−0.72, 1.18); 0.630	**7.85 (0.38, 15.33); 0.040**	**2.78 (0.00, 5.57); 0.050**	**1.04 (0.30, 1.79); 0.007**	5.20 (−15.12, 25.52); 0.610	5.70 (−8.76, 20.15); 0.433	−0.04 (−1.36, 1.28); 0.955	1.24 (−2.13, 4.61); 0.463	2.06 (−0.37, 4.49); 0.095	0.21 (−0.45, 0.88); 0.521
Sex, male	0.36 (−1.12, 1.84); 0.627	0.16 (−0.68, 0.99); 0.711	−2.12 (−8.73, 4.49); 0.522	−0.71 (−3.18, 1.75); 0.563	−0.29 (−0.95, 0.37); 0.378	3.41 (−14.55, 21.37); 0.705	−3.16 (−15.94, 9.61); 0.622	0.26 (−0.90, 1.43); 0.653	1.65 (−1.32, 4.63); 0.270	−1.31 (−3.45, 0.84); 0.227	**0.62 (0.04, 1.21); 0.038**
Age, years	−0.05 (−0.14, 0.04); 0.260	−0.03 (−0.07, 0.02); 0.307	0.02 (−0.37, 0.40); 0.933	0.00 (−0.14, 0.15); 0.975	0.00 (−0.04, 0.04); 0.928	0.36 (−0.69, 1.41); 0.496	0.08 (−0.67, 0.83); 0.832	0.01 (−0.05, 0.08); 0.662	−0.04 (−0.22, 0.13); 0.624	−0.03 (−0.15, 0.10); 0.660	−0.01 (−0.05, 0.02); 0.529
Adherence, %	0.05 (−0.25, 0.34); 0.751	−0.00 (−0.17, 0.16); 0.979	**−1.39 (−2.71, −0.08); 0.038**	−0.33 (−0.82, 0.16); 0.177	**−0.14 (−0.27, −0.01); 0.035**	1.08 (−2.48, 4.64); 0.546	0.18 (−2.36, 2.71); 0.890	−0.08 (−0.31, 0.15); 0.486	−0.22 (−0.81, 0.37); 0.452	0.12 (−0.31, 0.55); 0.573	0.10 (−0.01, 0.22); 0.085
Marginal R^2	0.608	0.782	0.518	0.578	0.682	0.612	0.563	0.475	0.440	0.253	0.453
Conditional R^2	0.965	0.873	0.962	0.867	0.887	0.877	0.943	0.836	0.861	0.955	0.897

Abbreviations and units: BMI, body mass index (kg/m^2); PASI, Psoriasis Area and Severity Index; Glucose (mg/dL); insulin (μIU/mL); HOMA-IR, Homeostatic Model Assessment for Insulin Resistance; TG, triglycerides (mg/dL); TC, total cholesterol (mg/dL); CRP, C-reactive protein (mg/L); ESR, erythrocyte sedimentation rate (mm/h); Urea (mg/dL); SUA, serum uric acid (mg/dL); T2DM, type 2 diabetes mellitus; NAFLD, non-alcoholic fatty liver disease. CI, confidence interval; R^2, coefficient of determination.

Figure S1. Predicted PASI scores at baseline and after 18 months according to weekly dose. Points represent model-estimated marginal means, and error bars indicate 95% confidence intervals. Estimates were obtained from a linear mixed-effects model with a random intercept for patient.

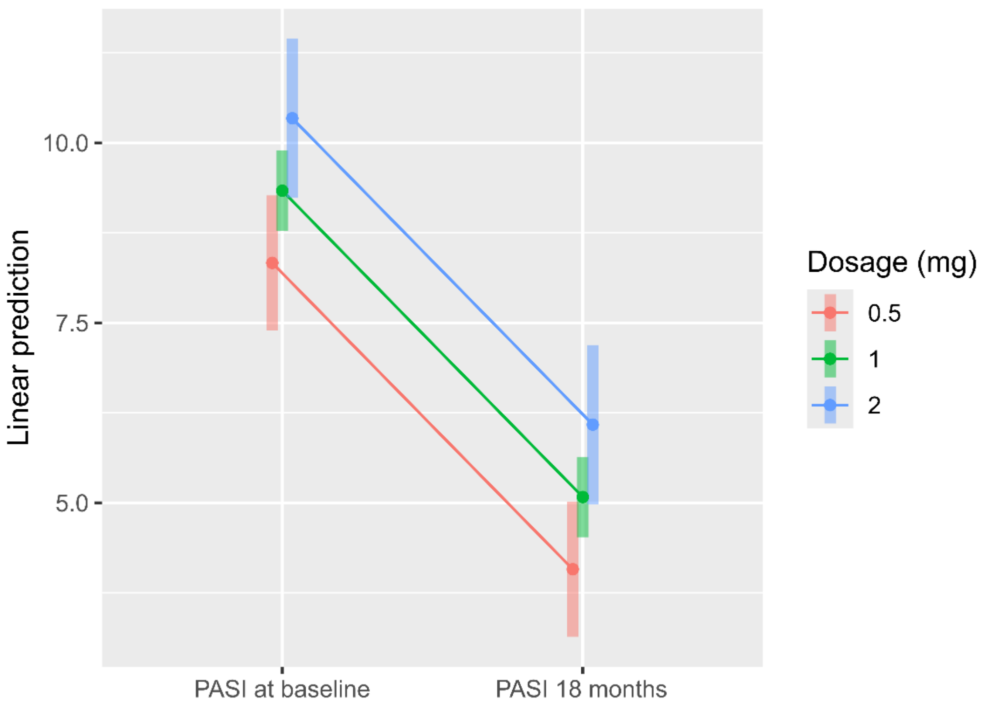

Supplement: Supplementary file 1 [file jcm-15-08015-s001.zip › Supplementary material_03.09.2026.pdf]
